# The impact of immunocompromise on outcomes of COVID-19 in children and young people—a systematic review and meta-analysis

**DOI:** 10.3389/fimmu.2023.1159269

**Published:** 2023-08-25

**Authors:** James Greenan-Barrett, Samuel Aston, Claire T. Deakin, Coziana Ciurtin

**Affiliations:** ^1^Department of Adolescent Rheumatology, University College London Hospital (UCLH), London, United Kingdom; ^2^Medical School, University College London (UCL), London, United Kingdom; ^3^Centre for Adolescent Rheumatology Versus Arthritis at UCL, UCLH and Great Ormond Street (GOS) Hospital (GOSH), London, United Kingdom; ^4^UCL GOS Institute of Child Health, UCL, London, United Kingdom; ^5^Department of Paediatric Rheumatology GOSH, London, United Kingdom; ^6^National Institute of Health Research - Biomedical Research Centre, UCLH, London, United Kingdom

**Keywords:** COVID - 19, immunosuppression, ITU - (intensive therapy unit), hospitalization, death, children, young people

## Abstract

**Background:**

Despite children and young people (CYP) having a low risk for severe coronavirus disease 2019 (COVID-19) outcomes, there is still a degree of uncertainty related to their risk in the context of immunodeficiency or immunosuppression, primarily due to significant reporting bias in most studies, as CYP characteristically experience milder or asymptomatic COVID-19 infection and the severe outcomes tend to be overestimated.

**Methods:**

A comprehensive systematic review to identify globally relevant studies in immunosuppressed CYP and CYP in general population (defined as younger than 25 years of age) up to 31 October 2021 (to exclude vaccinated populations) was performed. Studies were included if they reported the two primary outcomes of our study, admission to intensive therapy unit (ITU) and mortality, while data on other outcomes, such as hospitalization and need for mechanical ventilation were also collected. A meta-analysis estimated the pooled proportion for each severe COVID-19 outcome, using the inverse variance method. Random effects models were used to account for interstudy heterogeneity.

**Findings:**

The systematic review identified 30 eligible studies for each of the two populations investigated: immunosuppressed CYP (*n* = 793) and CYP in general population (*n* = 102,022). Our meta-analysis found higher estimated prevalence for hospitalization (46% vs. 16%), ITU admission (12% vs. 2%), mechanical ventilation (8% vs. 1%), and increased mortality due to severe COVID-19 infection (6.5% vs. 0.2%) in immunocompromised CYP compared with CYP in general population. This shows an overall trend for more severe outcomes of COVID-19 infection in immunocompromised CYP, similar to adult studies.

**Interpretation:**

This is the only up-to-date meta-analysis in immunocompromised CYP with high global relevance, which excluded reports from hospitalized cohorts alone and included 35% studies from low- and middle-income countries. Future research is required to characterize individual subgroups of immunocompromised patients, as well as impact of vaccination on severe COVID-19 outcomes.

**Systematic Review Registration:**

PROSPERO identifier, CRD42021278598.

## Introduction

Coronavirus disease 2019 (COVID-19) caused by the respiratory virus SARS-CoV-2 has, to date, resulted in over 600 million confirmed cases and 6.5 million deaths (1). However, children and young people (CYP), defined by the World Health Organization as aged 0–24 years (2), remain at low risk for severe outcomes of COVID-19 infection such as hospitalization, admission to intensive therapy units (ITUs), and death, reported in a large international meta-analysis as just 3.3%, 0.3%, and 0.02%, respectively (3).

The role of the immune system in SARS-CoV-2 transmission, clearance, and disease severity and the impact of immunocompromised states on COVID-19 infection severity in CYP are not entirely clear as more research has been directed toward investigating adult immunosuppressed populations (4, 5) especially as COVID-19 infection is associated with significantly poorer outcomes in older adults. However, it has already been established in large population studies that older adolescents and young adults, as well as the ones from ethnic minorities and with underlying medical conditions, are at risk for more severe outcomes following infection with SARS-CoV-2 (6).

A better understanding of the impact of immunocompromise on the severity of COVID-19 is important for risk stratification to guide strategies for administration of COVID-19 vaccines and therapeutics, immunosuppressive treatment management during infection and immunisation, as well as wider public health policies.

Immunocompromise has been investigated as an independent risk factor for COVID-19 in CYP, and studies have shown that immunocompromised patients are over-represented in cohorts of patients admitted to ITU or receiving invasive ventilation, 23% and 17%, respectively (7–9). Identifying the impact of immunocompromise on COVID-19 disease severity in CYP is challenging. Studies tend to have low sample sizes due to low prevalence of COVID-19 infection in CYP and were heterogeneous in relation to the type and severity of immunocompromise. In addition, the low rates of COVID-19 infection in immunocompromised CYP, likely due to shielding, and the low rates of SARS-CoV-2–related complications in CYP overall pose challenges for reaching the statistical certainty needed to draw definite conclusions. In CYP, non-specific symptoms, asymptomatic carriage of the virus, and variation in testing, in addition to bias in retrospective data collection and exclusive inclusion of hospitalized patients, may result in over-estimation of severity of COVID-19 infection.

A single meta-analysis investigating comorbidities associated with severe COVID-19 infection in children (defined as requiring ITU admission or invasive ventilation or resulting in death) demonstrated significantly higher rates of severe infection in immunocompromised children compared with general population controls (17.5% vs. 11.0%; RR, 1.44; 95% CI, 1.01–2.04), although there was no significant difference in disease severity in subgroups of hemato-oncology patients, patients on immunosuppressant drugs, or mixed immunosuppression when compared with general population controls (10). This meta-analysis included 154 immunocompromised children from 10 studies performed in Europe/USA, of which five studies included only hospitalized children with COVID-19.

### Objective

This paper addresses the need for a more comprehensive meta-analysis with global relevance based on an updated systematic review of the literature, aiming to minimize the risk for selection bias by including reports from low- and middle-income countries (LMICs), as well as studies not exclusively focused on hospitalized patients, in order to ensure the relevance of findings for children all over the world.

## Methods

### Search strategy and selection criteria

This is a systematic review and meta-analysis that was performed in accordance with the Preferred Reporting Items for Systematic Reviews and Meta-Analyses (PRISMA) guidelines (11). The review protocol was registered with the International Prospective Register of Systematic Reviews (PROSPERO - CRD42021278598). To identify studies on immunocompromised CYP, we performed a systematic search of the literature from 31 December 2019 to 31 October 2021 (prior to the widespread of rollout of COVID-19 vaccination in CYP) in the electronic databases PubMed and Scopus using MeSH terms COVID-19, child, infant, adolescent, pediatric, young adult, immunosuppressant, immunosuppression, immunocompromised, and immunologic deficiency syndrome (**Supplementary Table S1**). To identify studies capturing CYP in general population, we first performed a search of the literature for pre-existing systematic reviews on COVID-19 in CYP and identified a study published by Ifran et al, which included studies from 1 December 2019 to 8 January 2021 (12). We then performed a systematic search of the literature from 8 January 2021 to 31 October 2021 in PubMed and Scopus to update it. We identified other relevant studies by searching Google Scholar and reviewing the references of included studies (snowballing) (**Supplementary Table S2**). There were no restrictions on language, and reports that were not in English were translated using Google translate.

Eligible studies for inclusion were cohort or cross-sectional studies that included CYP under 25 years of age with COVID-19 infection, which reported the two primary outcomes of our study: admission to ITU and mortality in general population and in immunocompromised CYP. The definition of COVID-19 infection was based on either positive polymerase chain reaction (PCR) testing, antigen testing, serological testing, or if highly clinically suspected (based on compatible symptoms, radiology, and contact with confirmed case) to avoid reporting bias as access to PCR testing varied between countries and was restricted in the early pandemic. Where studies reported both adult and pediatric patients, the study was included only if it was possible to manually identify and remove patients aged 25 years or older. The immunocompromised CYP were defined as having chemotherapy or immunosuppressant therapy currently or within past 6 months, being post-haematopoietic stem-cell transplantation (HSCT) and on immunosuppression without reaching immune reconstitution, having primary immunodeficiency, bone marrow failure, sickle-cell disease, or being classified as immunocompromised.

Studies were excluded if they only reported data on patients admitted to hospital or ITU to avoid selection bias, as these cohorts would likely have poorer outcomes. Reports on oncology patients who were not immunocompromised (e.g., not on chemotherapy/immunosuppressants or patients who had immune reconstitution post-HRCT) or case studies of fewer than five patients were also excluded as not representative. Studies which only investigated age subgroups (e.g., neonates) were excluded to avoid skewing the results. Where reports overlapped, only the most recent study was included. Studies that included CYP who had received a vaccine to SARS-CoV-2 were excluded, and the search period was limited to the end of October 2021 to minimize inclusion of vaccinated CYP.

The identified studies were screened independently by two authors (J.G.B. and S.A.) based on titles and abstracts before full texts were screened. Studies were then selected for inclusion using the inclusion and exclusion criteria above and agreed on between both authors. Any disagreements were discussed with a third author (C.C.), and a consensus was agreed.

### Data analysis

Two authors (J.G.B. and S.A.) extracted data on epidemiological features, type of immunocompromise and outcomes (hospitalization, ITU admission, requirement for mechanical ventilation, and death) from eligible studies and entered it into a structured data extraction table. Where studies included patients aged 25 years or older, or not immunocompromised as per the aforementioned criteria, they were manually removed if per patient data was available. For general population CYP studies, if any immunocompromised patients were included, they were also manually removed where possible. Patients admitted to hospital or ITU for reasons unrelated to COVID-19 infection (e.g., routine chemotherapy) were excluded from the analysis. Where information of interest was not stated in the main paper or included in **Supplementary Data**, the corresponding authors were contacted. To evaluate study quality, we used the validated Newcastle-Ottawa scale (NOS) for cohort studies, which assesses the study selection, comparability, and outcomes (13)generating scores between 0 and 9, with 9 representing the highest study quality. Only one study included both immunocompromised and non-immunocompromised CYP (14).

The number of CYP with severe outcomes of COVID-19 infection in each study in immunocompromised versus general populations was used to evaluate the COVID-19–related morbidity (hospitalization, ITU admission, or mechanical ventilation requirement) and mortality and to compare outcomes. It was not possible to compare the overall estimated prevalence of outcomes of interest between the immunosuppressed CYP *versus* CYP in the general population or calculate the relative risk for each outcome as only one study reported data on both (14). The proportion of each outcome of interest was calculated for each study, in which, a continuity correction by adding 0.5 was applied when a study contained zero events.

A pooled proportion was then estimated for each outcome using meta-analysis, using the inverse variance method. Random effects models were used to account for interstudy heterogeneity, and studies were weighted according to their size and variance. Study-specific heterogeneity was assessed using the l (2) statistic (0%–100%), in which a lower value implies less heterogeneity. The between-study variance tau (2) was computed using the maximum likelihood method and tested for the assumption of homogeneity using the Wald test. The possibility of reporting bias influencing results was addressed through a combination of visual inspection of funnel plots for asymmetry, formal tests of bias using the weighted linear regression method (Egger’s test), and sensitivity analyses using the “trim-and-fill” method. All statistical analyses were carried out using the meta library version 4.14-0 in R version 4.0.2.

## Results

Only one eligible study concomitantly reported COVID-19 outcomes in immunosuppressed CYP *versus* CYP in general population (14).

### Systematic review of severe COVID-19 infection outcomes in immunosuppressed CYP

**Figure 1:**
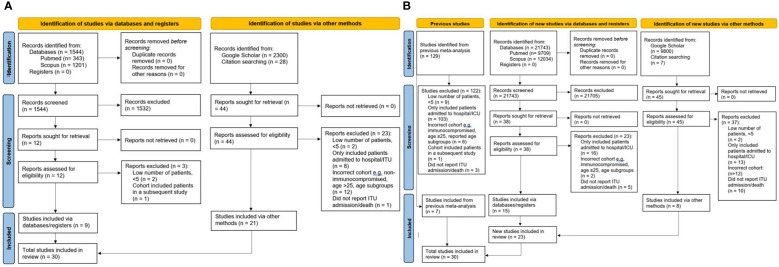
PRISMA flow charts for all studies in the systematic analysis. **(A)** studies of immunocompromised CYP **(B)** general population studies of CYP. CYP, children and young people; ITU, Intensive Therapy Unit.

The electronic search identified 1,544 studies of immunocompromised CYP, out of which 1,532 were excluded after screening, and another three after assessment of the eligibility criteria. Nine studies identified electronically, and 21 further studies identified manually from other sources were finally included in the systematic review (**Figure 1A**) (14–43).

The studies varied in size and included from five to 113 patients. In total, 793 immunocompromised CYP were included in our systematic review and meta-analysis (**Table 1**).

**Table 1:** Studies included in the systematic review of severe COVID-19 infection outcomes in immunosuppressed CYP.

Author	Country	Multicentre	Age	Immunodeficiency type	n	Admitted to hospital	Admitted to ITU	Required invasive ventilation	Died	NOS
**Antúnez-Montes**	South America	Yes	≤18	Mixed	28	25	7	5	4	6
**Bisogno**	Italy	Yes	<18	Haematology/oncology	29	12	0	0	0	6
**Castano‐Jaramillo**	Mexico	Yes	<25	Primary immunodeficiency	21	12	6	NR	4	6
**De Rojas**	Spain	No	≤18	Haematology/oncology	15	7	0	0	0	5
**Delavari**	Iran	Yes	<25	Primary immunodeficiency	16	16	8	NR	8	6
**Deya-Martinez**	Spain	No	≤22	Primary immunodeficiency	15	2	0	0	0	6
**El-Dannan**	UAE	No	≤18	Mixed	5	5	0	0	0	5
**Esenboga**	Turkey	No	<25	Primary immunodeficiency	15	7	0	0	0	4
**Faura**	Spain	Yes	≤18	Mixed	47	20	4	2	2	6
**Ferrari**	Italy	Yes	<18	Haematology/oncology	15	NR	1	1	0	6
**Gampel**	USA	Yes	≤21	Mixed	16	10	4	2	1	5
**Goss**	USA	Yes	≤18	Solid organ transplant	26	5	0	0	0	6
**Götzinger**	Europe	Yes	≤18	Mixed	57	48	3	1	1	5
**Hrusak**	Worldwide	Yes	<18	Haematology/oncology	8	NR	0	0	0	5
**Ihara**	Brazil	No	<18	Immunosuppressant	11	1	0	0	0	6
**Kamdar**	USA	No	<18	Mixed	64	21	7	6	2	6
**Lucchini**	UK	Yes	≤18	Haematology/oncology	6	NR	0	0	0	6
**Madhusoodan**	USA	Yes	≤21	Haematology/oncology	98	28	17	7	4	6
**Marcus**	Israel	Yes	<25	Primary immunodeficiency	13	0	0	0	0	6
**Marlais**	Worldwide	Yes	≤19	Immunosuppressant	113	68	6	5	4	6
**Melgosa**	Spain	Yes	<18	Immunosuppressant	9	NR	0	0	0	5
**Meyts**	Worldwide	Yes	<25	Primary immunodeficiency	36	25	6	6	2	6
**Millen**	UK	Yes	<16	Haematology/oncology	38	13	2	1	0	5
**Perez-Martinez**	Spain	No	≤18	Mixed	8	5	0	0	0	5
**Rao**	India	No	≤18	Mixed	16	8	6	1	2	6
**Rouger-Gaudichon**	France	Yes	<25	Haematology/oncology	37	20	5	2	1	5
**Singer**	USA	No	≤21	Solid organ transplant	5	1	0	NR	0	5
**Turner**	Worldwide	Yes	<18	Immunosuppressant	8	0	0	0	0	5
**Vicent**	Spain	No	≤18	Haematology/oncology	8	2	2	2	2	5
**Yuksel**	Turkey	No	<18	Solid organ transplant	10	3	0	NR	0	6

CYP, children and young people; ITU, Intensive Therapy Unit.

Of the 30 eligible studies on immunocompromised patients, 19 (63.3%) were multicenter, six (20.0%) were multinational, and 12 (40.0%) were from LMIC. Eight (26.7%) studies included patients with hematological or oncological malignancies on chemotherapy or immunotherapy, six (20.0%) included patients with primary immunodeficiency, three (10.0%) included patients with solid organ transplant (SOT) on immunosuppression, four (13.3%) included other patients on immunosuppression, and eight (26.7%) included a mix of immunocompromised patients. The age cutoffs for patient inclusion are detailed in **Table 1**.

In addition to the mandatory reported outcomes (ITU admission and death), 26 studies (86.7%) reported hospitalization and 26 studies (86.7%) reported invasive ventilation. The quality of the studies was poor moderate (NOS scores 4–6/9) (**Table 1**).

### Systematic review of severe COVID-19 infection outcomes in CYP in general population

The previous systematic review in CYP in general population by Ifran et al. identified 129 studies of which seven fulfilled inclusion criteria (12). The additional literature search we performed identified 21,743 studies in CYP in general population, of which 21,705 were excluded after screening, leading to 38 studies which were assessed for eligibility, out of which 15 studies were eligible for inclusion. Eight additional eligible studies were retrieved manually from other sources (**Figure 1B**). Our final analysis included 23 eligible studies identified by our searched and seven studies captured by the previous systematic analysis (14, 44–72).

The studies varied in size from 14 to 43,465 patients. In total, 102,022 non–immunocompromised patients were included in our systematic review and meta–analysis (**Table 2**).

**Table 2:** Studies included in the systematic review of severe COVID-19 infection in CYP in general population.

Author	Country	Multicentre	Age	n	Admitted to hospital	Admitted to ITU	Required invasive ventilation	Died	NOS
**Bailey**	USA	Yes	<25	5374	359	99	33	8	5
**Bayesheva**	Kazakhstan	Yes	≤18	650	7	6	3	0	5
**Chao**	USA	No	≤21	67	46	13	6	1	5
**Foster**	USA	Yes	<21	57	8	0	0	0	5
**Gotzinger**	Europe	Yes	≤18	525	NR	45	NR	3	5
**Howard**	USA	No	≤18	1000	41	8	2	1	4
**Katayama**	Japan	Yes	≤19	1240	150	1	1	0	5
**Kim**	USA	Yes	<18	2763	142	78	5	1	6
**Kompaniyets**	USA	Yes	≤18	43465	4302	1273	277	38	5
**Korkmaz**	Turkey	No	<18	81	37	2	NR	0	5
**Lavaine**	France	No	<18	33	26	0	0	0	4
**Lazzerini**	Italy	Yes	<18	190	48	2	0	0	6
**Lu**	China	No	<16	171	21	2	3	1	5
**Mania**	Poland	No	<18	106	12	0	0	0	6
**Matteudi**	France	Yes	<16	194	NR	0	0	0	4
**Meyer**	Germany	No	<18	65	9	1	NR	0	5
**O’Horo**	USA	Yes	<18	674	13	3	1	0	6
**Onal**	Turkey	No	<18	37	20	10	1	0	4
**Otto**	USA	Yes	≤21	424	51	25	12	2	5
**Parri**	Italy	Yes	≤18	170	115	4	1	0	5
**Pokorska-Śpiewak (Mar 21)**	Poland	No	≤18	15	4	0	0	0	5
**Pokorska-Śpiewak (Oct 21)**	Poland	Yes	≤18	1283	1008	3	0	0	5
**Qiu**	China	Yes	<17	36	NR	0	0	0	5
**Saatci**	UK	Yes	≤18	26322	343	73	NR	1	5
**Sarangi**	India	No	≤18	50	NR	0	0	0	5
**Schönfeld**	Argentina	Yes	≤18	13617	2094	118	NR	25	5
**Soriano-Arandes**	Spain	Yes	<16	1040	27	1	NR	0	5
**Wang**	China	Yes	<18	1369	NR	3	NR	1	5
**Yock‐Corrales**	South America	Yes	<18	990	303	47	31	8	5
**Yousaf**	USA	Yes	<18	14	0	0	0	0	4

CYP, children and young people; ITU, Intensive Therapy Unit.

Of the 30 included studies in CYP in general population, 19 were multicenter (63.3%), two (6.7%) were multinational, and nine (30%) were from LMIC. The age cutoffs are detailed in **Table 2**.

In addition to the mandatory reported outcomes (ITU admission and death), 25 studies (83.3%) reported hospitalization and 23 studies (76.7%) reported invasive ventilation. four studies (13.3%) were scored six stars in the NOS, 21 (70%) were scored five stars and five (16.7%) was scored four stars, out of a total of nine.

### Meta–analysis of severe COVID–19 infection outcomes in immunosuppressed CYP

There were 30 studies included in the meta–analysis (**Supplementary** **Tables S1, S3**).

**Figure 2:**
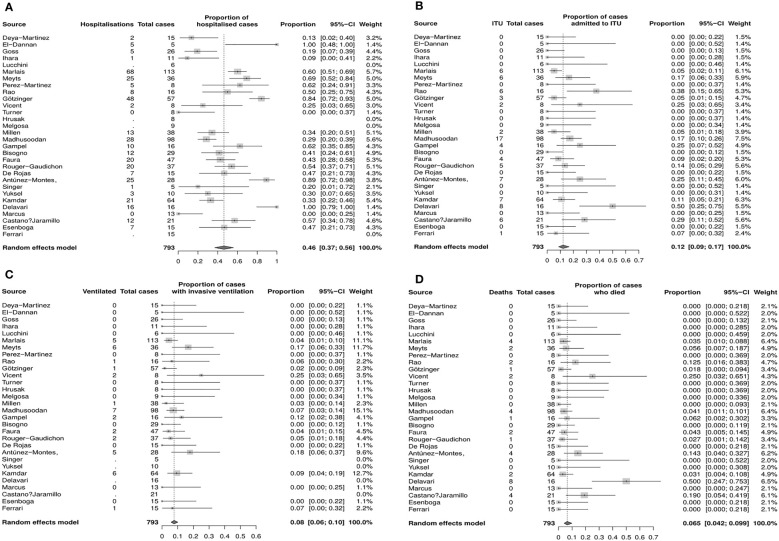
Estimated proportions of COVID–19 outcomes of interest in immunocompromised CYP (random effects model, 95% CI), **(A)** Hospitalization **(B)** Admission to ITU **(C)** Mechanical ventilation **(D)** Death. CI, confidence interval; CYP, children young people; ITU, intensice therapy unit.

The pooled proportion estimate for hospital admission due to COVID–19 infection was 46% (95% CI 37%–56%, **Figure 2A**) and for ITU admission due to severe COVID–19 infection was 12.0% (95% CI 9%–17%, **Figure 2B**). The estimated proportion of patients that required invasive ventilation was 8% (95% CI 6%–10%, **Figure 2C**), and the mortality rate was estimated to be 6.5% (4.2%–9.9%, **Figure 2D**).

Funnel plots and sensitivity analyses indicated that the proportion admitted to hospital was unlikely to be affected by reporting bias, however, the estimated proportion of patients admitted to ITU or requiring invasive ventilation and mortality rate may be significantly affected by bias (**Supplementary Figure S1**).

### Meta–analysis of severe COVID–19 infection outcomes in CYP in general population

There were 30 studies included in the meta–analysis (**Supplementary** **Tables S2, S3**).

**Figure 3:**
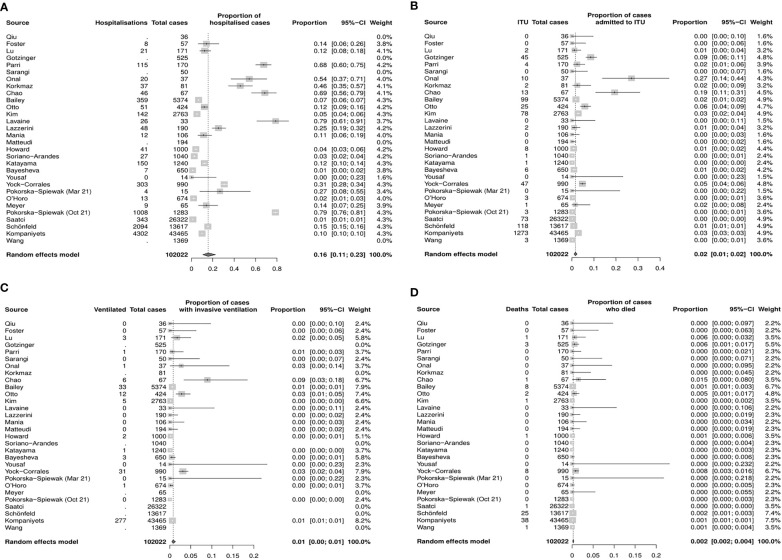
Estimated proportions of Covid–19 outcomes of interest inCYP in the general population (random effects model, 95% CI) **(A)** Hospitalization **(B)** Admission to ITU **(C)** Mechanical ventilation **(D)** Death. CI, confidence interval; CYP, children and young people; ITU, intensive therapy unit.

The pooled proportion estimate for hospital admission due to COVID–19 infection was 16% (95% CI 11%–23%, **Figure 3A**), while the estimate for ITU admission due to severe COVID–19 infection was 2.0% (95% CI 1%–2%, **Figure 3B**). The proportion of CYP who required mechanical ventilation requirements was 1% (95% CI 0%–1%, **Figure 3C**), and the mortality rate was estimated to be 0.2% (95% CI 0.2%–0.4%, **Figure 3D**).

Funnel plots and sensitivity analyses indicated that the proportion admitted to hospital, requiring ITU or invasive ventilation, was unlikely to be affected by reporting bias, however, the mortality rate may be significantly affected by bias (**Supplementary Figure S2**).

## Discussion

This is the most up–to–date systematic review and meta–analysis of outcomes of severe COVID–19 infection in CYP and the only one assessing immunosuppressed non–hospitalized cohorts in comparison with general population.

Our meta–analysis found higher estimated prevalence for hospitalization (46% vs. 16%), ITU admission (12% vs. 2%), mechanical ventilation (8% vs. 1%), and increased mortality due to severe COVID–19 infection (6.5% vs. 0.2%) in immunocompromised CYP compared with CYP in general population. This shows an overall trend for more severe outcomes of COVID–19 infection in immunocompromised CYP, such as found in adults. Comparisons with published literature are influenced by the type of populations analyzed (adults vs. children) as well as setting (hospitalized cohorts vs. general population cohorts). Adult rheumatology registry studies have shown that glucocorticoids or cyclophosphamide treatment increased the risk of severe COVID–19 outcomes (73, 74). Cohort studies of 13,206 Spanish and 6,435 Korean patients demonstrated statistically significant higher inpatient mortality in immunocompromised compared with non–immunocompromised patients, 31.3% *versus* 19.3% and 6.4% *versus* 2.0%, respectively (75, 76). A meta–analysis of 2,777 pediatric and adult SOT patients showed very high rates of hospitalization (81%), ITU admission among hospitalized patients (29%) and mortality (18.6%) (77). A meta–analysis comparing both pediatric and adult SOT patients hospitalized with COVID–19 to the general population demonstrated significantly higher rates of ITU admission (35.8% vs. 23.1%) and mortality (23.2% vs. 12.5%) (4). Another meta–analysis also found higher mortality from COVID–19 in oncology patients on chemotherapy (OR: 1.85, 95% CI: 1.26–2.7) but no difference in oncology patients on immunotherapy (78).

There are several studies that found lower rates of severe COVID–19 outcomes. A prospective cohort of immunocompromised children in the UK found that 4 of 38 children (10.5%) with COVID–19 were hospitalized, with no cases requiring ITU admission or resulting in death, with the caveat that the outcomes were self–reported and the sample size was small (79). A meta–analysis comparing risks of severe COVID–19 infection in immunodeficient and immunosuppressed pediatric and adult patients to general population did not demonstrate statistically significant differences, although included only 28 immunodeficient and 11 immunosuppressed patients (80). A cohort study of Italian children and adults with primary immunodeficiencies did not demonstrate statistically different rates of COVID–19 mortality compared with the general population (3.81% vs. 3.28%), although the mortality rates in this study were significantly higher than in other population based cohorts (81).

Immunocompromised patients are heterogeneous and include a wide variety of suppressed or defective immune system responses. The innate immune system may be affected in primary immunodeficiencies (type I interferon response abnormalities) or in the context of immunosuppressive therapies (e.g., glucocorticosteroids, which inhibit the macrophage function, biologic therapies, which block pro–inflammatory cytokines, or small molecule targeting transcription factors implicated in the innate immune cell responses). The detection of viral RNA by dendritic cells using toll–like receptors, leading to subsequent interferon signaling is postulated to be vital in the early defence to SARS–CoV–2, and this response can be significantly altered in the context of immunodeficiency or immunosuppression. The adaptive immune response can be affected by other forms of immunocompromise (e.g., various B– and T–cell primary immunodeficiencies or iatrogenic immunosuppression affecting B– and T–cell function) and is important in clearing SARS–CoV–2 infection as well as regulating the overall immune response to infection. A good cytotoxic CD8+ T–cell response is thought to be important for early viral clearance, while memory T cells and B cells are vital for developing protective immunity after infection or vaccination (82).

This meta–analysis has several strengths. It is the only up–to–date meta–analysis in CYP that addressed the risk of selection bias by excluding papers reporting only on hospitalized cohorts. It is a large meta–analysis, including 793 immunocompromised and 102,022 non–immunocompromised CYP from a total of 60 studies, many of which were multicenter and multinational. Additionally, this meta–analysis included many studies from outside Europe/USA and 35% of studies were from LMIC, therefore, the results are likely relevant to immunocompromised CYP globally. A particular strength of this study was the stringent inclusion criteria and selection of truly immunocompromised CYP and exclusion vaccination as major confounder. The sensitivity analysis showed low risk of bias in reporting hospitalization in both groups, as well as ITU admission and mechanical ventilation in CYP in the general population.

This meta–analysis also has limitations, which suggest a need for cautious interpretation of our findings. The estimated prevalence of severe outcomes of COVID–19 infection in CYP in general population from our analyses is higher than outcomes from surveillance studies, for example, a UK national database study found 2.7% of children with confirmed COVID–19 were hospitalized (83), compared with 16% in our study. This difference is likely due to differences in the study settings, with lower rates of severe disease in “community–based studies” that use public health reporting systems compared with “healthcare–based studies” that recruit COVID–19 patients who present to healthcare services. This disparity was demonstrated in another meta–analysis that found that rates of hospitalization, ITU admission and death from COVID–19 in children in community–based general population studies were 3.3%, 0.3%, and 0.1%, respectively, which was strikingly different from findings in healthcare–based studies (23.9%, 2.9%, and 1.3%, respectively) (3), which matches the estimates from our meta–analysis. The explanation for this may be that asymptomatic or mildly symptomatic patients or who have less severe COVID–19 infection are underrepresented in healthcare–based studies. In our meta–analysis, most studies were healthcare based as many community–based studies did not report both ITU admission and death and the studies were quite heterogeneous, reporting variable estimates of severe COVID–19 outcomes, which may explain our higher–than–expected rates of severe outcomes of COVID–19 infection in non–immunocompromised CYP. Although the quality of outcome reporting in some studies led to a degree of selection bias, importantly, the same process of study selection was used for both the immunocompromised and non–immunocompromised meta–analyses in our study.

Retrospective study designs and clinician reporting of cases (especially in multicenter studies) will also result in reporting bias due to over–representation of severe cases. Additionally, although the non–immunocompromised CYP in the general population studies included in this analysis were carefully selected to avoid inclusion of immunocompromised CYP, they may have had other comorbidities that we could not account for.

There were large variations in COVID–19 clinical practice between healthcare centers, between countries, and over time, including variations in indications for testing, with some studies capturing populations tested only if symptomatic and unable to account for mild or asymptomatic cases, which are very common in CYP. There were also variations in practice in relation to hospital/ITU admission, as well as potentially more cautious approaches taken for immunocompromised patients (e.g., admitting them to hospital for monitoring), while more objective outcomes, such as rates of ventilation and death were less likely to be affected.

Additionally, the impact of COVID–19 therapeutics on outcomes of disease is unknown as this was variably reported. None of the patients included in this meta–analysis was reported to be vaccinated, as assessing the impact of immunocompromise on vaccinated CYP was beyond the scope of this paper. This analysis covers different waves of the pandemic in different countries, with variable access to treatments for which we could not account for, although there is some evidence for stable outcomes in COVID–19 outcomes in hospitalized patients over time (84).

Although this study demonstrated an overall trend for more severe outcomes of COVID–19 infection in immunocompromised CYP, it is not possible to attribute causality solely to the immunocompromise itself as most patients with have co–existent comorbidities, such as additional genetic abnormalities or comorbidities. Additionally, there is likely significant inter– and intra–group heterogeneity within immunocompromised cohorts (e.g., differences in types and doses of immunosuppressive medications), which may influence COVID–19 outcomes and was not possible to investigate in this analysis.

Future research must investigate the risk of severe COVID–19 in individual subgroups of immunocompromised patients and establishing the impact of immunosuppression type or dose as well as that of various comorbidities and other factors, such as age, sex, ethnicity, and socioeconomic status on COVID–19 risk. Further research is needed to investigate the efficacy of vaccination in preventing COVID–19 infection and the role of various therapeutics in treating COVID–19 infection in subgroups of immunocompromised CYP to support evidence–based recommendations for risk stratification and tailored management. Finally, it is vital that we continue to investigate how COVID–19 interacts with the immune system and the biological mechanism by which immunosuppression affects viral replication.

## Data availability statement

The original contributions presented in the study are included in the article/**Supplementary Material**. Further inquiries can be directed to the corresponding author.

## Author contributions

Design of the study, JG–B, CC. Electronic searches and paper screening, JG–B, SA. Study selection and inclusion, JG–B, SA, CC. Data extraction and analysis for the systematic review, JG–B, SA, CC. Meta–analysis, CD. Writing the manuscript, JG–B, CC, CD: Review of the manuscript, CC, JG–B, CD. All authors approved the final version of the manuscript.
