# Supplementary material for: The impact of immunocompromise on outcomes of COVID-19 in children and young people—a systematic review and meta-analysis
Source: Front Immunol. 2023 Aug 25;14:1159269. doi: 10.3389/fimmu.2023.1159269 (PMC10485615; doi:10.3389/fimmu.2023.1159269)
Supplement: Supplementary file 1 [file DataSheet_1.pdf]

Supplementary material

The impact of immunocompromise on outcomes of COVID-19 in children and young people - a systematic review and meta-analysis

James Greenan-Barrett[1], Samuel Aston[2], Claire T. Deakin[1,3]*, Coziana Ciurtin[1*#]

[1]Centre for Adolescent Rheumatology Research, Division of Medicine, University College London, Rayne Building, London WC1E 6JF, UK
[2]Medical School, University College London, Gower Street, London WC1E 6BT, UK
[3]Great Ormond Street Institute of Child Health, University College London, 30 Guilford Street, London WC1N 1EH, UK

Author	Study type	Method of COVID-19 diagnosis	Details on immunodeficiency	n	Admitted to hospital	Admitted to ITU	Required invasive ventilation	Required vasopressor	Died	Notes
**Antúnez-Montes †**	Retrospective & prospective	PCR / MIS-C criteria & serology	Immunosuppressant & malignancy on chemotherapy	28	25	7	5	5	4	
**Bisogno**	Prospective	PCR	Leukaemia [16], lymphoma [3] & other malignancy [10] on chemotherapy [25] or immunosuppressants [4]	29	12*	0	0	0	0	* 3 additional patients were hospitalised for reasons not related to COVID-19 and were manually excluded
**Castano-Jaramillo**	Retrospective	PCR / antigen / serology / high clinical suspicion	Mixed primary immunodeficiency	21*	12	6	NR	NR	4	* 10 additional patients were ≥25 and so were manually excluded
**De Rojas**	Case series	PCR	Haematology/oncology malignancy on chemotherapy & post HSCT on immunosuppressants	15	7*	0	0	0	0	* 4 additional patients were hospitalised for reasons not related to COVID-19 and were manually excluded
**Delavari**	Prospective	PCR	Mixed primary immunodeficiency – most common SCID	16*	16	8	NR	NR	8	* 3 additional patients were ≥25 and so were manually excluded
**Deya-Martinez**	Prospective	PCR / serology	Mixed primary immunodeficiency	15	2	0	0	0	0	
**El-Dannan**	Case series	PCR	CVID [1], malignancy on chemotherapy [1], pyruvate kinase deficiency post splenectomy [1], nephrotic syndrome on steroids [1] & SLE on immunosuppressants [1]	5	5	0	0	0	0	
**Esenboga**	Retrospective	PCR	Mixed primary immunodeficiency	15*	7	0	0	0	0	* 11 additional patients were ≥25 and so were manually excluded
**Faura †**	Retrospective	PCR	Non-malignant haematology (AIHA, sickle cell, bone marrow failure, primary immune thrombocytopenia, HLH) on immunosuppressants [6], malignancy [14] or leukaemia/lymphoma [19] on chemotherapy/ immunosuppressants & post HSCT on immunosuppressants [8]	47	20*	4	2	1	2	* 12 additional patients were hospitalised for reasons not related to COVID-19 and were manually excluded
**Ferrari**	Prospective	PCR	Haematology/oncology malignancy on chemotherapy	15*	NR	1	1	NR	0	* 6 additional patients had completed chemotherapy and were being followed up so were manually excluded
**Gampel**	Retrospective	PCR	Haematology/oncology malignancy on chemotherapy/immunosuppressants [13] & sickle cell [3]	16*	10	4	2	1	1	* 3 additional patients had haematological malignancies but were not on immunosuppressing therapy up so were manually excluded
**Goss**	Prospective	PCR	Kidney [8], liver [10], heart [6] & lung [2] transplant on immunosuppressants	26	5	0	0	0	0	
**Götzinger †**	Prospective	PCR	On immunosuppressant [29], primary immunodeficiency [3] & malignancy on chemotherapy [25]	57	48	3*	1*	0*	1	* 2 additional patients were admitted to ITU for reasons not related to COVID-19 as classified by the study contributors and they were manually excluded

**Hrusak**	Retrospective	NR	Haematology/oncology malignancy on chemotherapy	8	NR	0	0	0	0	
**Ihara**	Case series	PCR / serology	Rheumatological diseases on immunosuppressants	11*	1	0	0	0	0	* 3 additional patients were not on immunosuppression so were manually excluded
**Kamdar**	Retrospective	PCR	Sickle cell [30] & haematology/oncology malignancy on chemotherapy/immunosuppressants [34]	64*	21	7	6	NR	2**	* 45 additional patients had malignancy but were not on immunosuppressing therapy or were 100+ days post HSCT with count recovery and were not on immunosuppressants so were manually excluded ** 2 additional patients died but the study authors did not attribute the death to COVID-19 and they were manually excluded
**Lucchini**	Prospective	PCR	Post HSCT on immunosuppressant	6*	NR	0	0	0	0	* 3 additional patients were not on immunosuppressing therapy or were post HSCT with count recovery and were not on immunosuppressants so were manually excluded
**Madhusoodan**	Retrospective	PCR	Haematology/oncology malignancy on chemotherapy/ immunosuppressants [90] & post HSCT on immunosuppressant [8]	98	28*	17	7	NR	4	* 22 additional patients were hospitalised for reasons not related to COVID-19 and were manually excluded
**Marcus**	Retrospective	PCR / high clinical suspicion	Mixed primary immunodeficiency – most common SCID	13*	0	0	0	0	0	* 7 additional patients were ≥25 and so were manually excluded
**Marlais**	Prospective	PCR / serology / high clinical suspicion	Renal disease on immunosuppressant	113	68	6	5	NR	4	
**Melgosa**	Retrospective	PCR	Renal disease on immunosuppressant	9	NR	0	0	0	0	
**Meyts**	Retrospective	PCR / serology	Mixed primary immunodeficiency	36	25	6	6*	NR	2	* Includes 1 patient who required ECMO
**Millen †**	Retrospective & prospective	PCR	Haematology/oncology malignancy on chemotherapy/immunosuppressants	38*	13**	2	1	0	0***	* 16 additional patients had cancer diagnosis but information on treatment was not available and so were manually excluded ** 18 additional patients were hospitalised for reasons not related to COVID-19 and were manually excluded *** 1 additional patient died but study contributors did not attribute the death to COVID-19 and they were manually excluded
**Perez-Martinez**	Retrospective	PCR	HSCT on immunosuppression [3], leukaemia on chemotherapy [1], malignancy on immunotherapy [1], solid organ transplant on immunosuppressant [2] & rheumatological disease on immunosuppressant [1]	8	5	0	0	0	0	

**Rao †**	Retrospective	PCR	Renal disease on immunosuppressants [8], haematology malignancy on chemotherapy [8]	16	8	6	1	2	2	
**Rouger-Gaudichon**	Retrospective & prospective	PCR / serology & high clinical suspicion	Haematology/oncology malignancy on chemotherapy/immunosuppressants & post HSCT on immunosuppressant	37	20	5	2	NR	1	
**Singer**	Retrospective	PCR / serology	Renal transplant on immunosuppressant	5	1	0	NR	NR	0	
**Turner**	Retrospective	PCR / high clinical suspicion	Inflammatory bowel disease on immunosuppressant	8	0	0	0	0	0	
**Vicent †**	Case series	PCR	Post HSCT on immunosuppressant [5] or not count recovered [3]	8*	2	2	2	2	2**	* 3 additional patients were hospitalised for reasons not related to COVID-19 and were manually excluded ** Includes 1 patient who died after publication but was identified after contacting authors
**Yuksel †**	Retrospective	PCR / high clinical suspicion	Liver transplant on immunosuppressant	10	3	0	NR	NR	0	

Supplementary Table 1: Details of the study design, COVID-19 diagnostic tests used, type of immunosuppression and number of CYP with severe COVID-19 infection outcomes reported by all the immunosuppressed CYP studies included in the systematic review and meya-analysis.

Legend: AIHA- autoimmune haemolytic anaemia; CVID- common variable immunodeficiency; ECMO- extracorporeal membrane oxygenation; HLH- hemophagocytic lymphohistiocytosis; HSCT- haemopoietic stem cell transplant; ITU intensive therapy unit; MIS-C- multisystem inflammatory syndrome in children; NR- not recorded; PCR-polymerase chain reaction; SCID- severe combined immunodeficiency; SLE- systemic lupus erythematosus

† - the study author was contacted and provided more information than what was available in the manuscript

Author	Study type	Method of COVID-19 diagnosis	n	Admitted to hospital	Admitted to ITU	Required invasive ventilation	Required vasopressor	Died	Notes
**Bailey**	Retrospective	PCR	5374	359	99	33	NR	8	
**Bayesheva †**	Retrospective	PCR	650	7	6	3	NR	0	
**Chao**	Retrospective	PCR	67*	46	13	6	2	1	* Includes 2 patients who were immunocompromised – it was not possible to manually exclude them
**Foster**	Retrospective	PCR	57	8	0	0	0	0	
**Gotzinger †**	Prospective	PCR	525*	NR	45	NR	NR	3	* 57 additional patients were immunosuppressed so were manually excluded.
**Howard**	Prospective	PCR	1000*	41	8	2	NR	1	* Includes 9 patients who were immunocompromised – it was not possible to manually exclude them
**Katayama**	Retrospective	PCR	1240	150	1	1	NR	0	
**Kim**	Retrospective	PCR	2763	142	78	5	NR	1	
**Kompaniyets**	Cross-sectional	NR	43465	4302	1273	277	NR	38	
**Korkmaz**	Retrospective	PCR	81	37	2	NR	NR	0	
**Lavaine**	Retrospective	PCR	33	26	0*	0	0	0	* 1 additional patient was admitted to ITU for reasons not related to COVID-19 as classified by the study contributors and they were manually excluded
**Lazzerini**	Cross-sectional	PCR	190	48	2	0	0	0	
**Lu**	Prospective	PCR	171	21	2	3	NR	1	
**Mania**	Prospective	PCR	106	12	0	0	0	0	
**Matteudi**	Prospective	PCR	194	NR	0	0	0	0	
**Meyer**	Retrospective	PCR	65	9*	1	NR	NR	0	* 3 additional patients were hospitalised for reasons not related to COVID-19 as classified by the study contributors and they were manually excluded
**O'Horo †**	Retrospective	PCR	674	13	3	1	0	0	
**Onal**	Prospective	PCR / high clinical suspicion	37	20	10	1	NR	0	

**Otto**	Retrospective	PCR	424*	51	25	12	13	2	* Includes 14 patients who were immunocompromised – it was not possible to manually exclude them
**Parri**	Retrospective	PCR	170	115	4	1	NR	0	
**Pokorska-Śpiewak (Mar 21) †**	Prospective	PCR	15	4*	0	0	0	0	* 7 additional patients were hospitalised <1 day for the purpose of assessment/investigation and were manually excluded
**Pokorska-Śpiewak (Oct 21) †**	Prospective	PCR / antigen	1283	1008	3	0	0	0	
**Qiu**	Retrospective	PCR	36	NR	0	0	0	0	
**Saatci**	Retrospective	PCR	26322	343	73	NR	NR	1	
**Sarangi**	Prospective	PCR	50	NR	0	0	0	0	
**Schönfeld**	Cross-sectional	PCR	13617	2094	118	NR	NR	25	
**Soriano-Arandes †**	Prospective	PCR / antigen	1040	27	1	NR	NR	0	
**Wang**	Cross-sectional	PCR / high clinical suspicion	1369	NR	3	NR	NR	1	
**Yock-Corrales †**	Retrospective and prospective	PCR / serology with compatible history	990	303	47	31	39	8	
**Yousaf**	Prospective	PCR	14	0	0	0	NR	0	

Supplementary Table 2: Details of the study design, COVID-19 diagnostic tests used, and number of CYP with severe COVID-19 infection outcomes reported by all the CYP studies in the general population included in the systematic review and meta-analysis.

Legend: ICU- intensive care unit; NR- not recorded; PCR- polymerase chain reaction

† - the study author was contacted and provided more information than what was available in the manuscript

Outcome	Estimate	Lower 95% CI	Upper 95% CI	tau^2	I^2	Number of 'studies' added by trim-and-fill	Estimate (trim-and-fill)	Lower 95% CI (trim-and-fill)	Upper 95% CI (trim-and-fill)	P-value for asymmetry test
**Immunosuppressed CYP**										
Hospitalisation	0.1566	0.1059	0.2252	1.2173 [0.9797; 5.3624]	99.6% [99.5%; 99.6%]	4	0.0995	0.0631	0.1536	0.5365
ITU admission	0.0157	0.0104	0.0237	0.9262 [0.6265; 3.4910]	96.6% [95.8%; 97.2%]	9	0.0289	0.0191	0.0434	0.3032
Ventilation	0.0082	0.0049	0.0136	0.8298 [0.3340; 2.9250]	86.5% [81.0%; 90.4%]	0	0.0082	0.0049	0.0136	0.6283
Death	0.0025	0.0015	0.0041	0.8183 [0.2966; 2.8024]	68.3% [53.8%; 78.2%]	7	0.0016	0.001	0.0026	0.08081
**CYP in the general population**										
Hospitalisation	0.4635	0.3695	0.5602	0.6531 [0.4880; 2.3228]	78.3% [68.7%; 84.9%]	2	0.4846	0.3886	0.5818	0.7297
ITU admission	0.1221	0.0873	0.168	0.4156 [0.0000; 1.1444]	49.3% [22.5%; 66.8%]	11	0.1646	0.1204	0.2211	0.007431
Ventilation	0.0769	0.0582	0.101	tau^2 = 0 [0.0000; 0.1999]	0.0% [0.0%; 35.6%]	9	0.0895	0.069	0.1153	0.01559
Death	0.0647	0.0419	0.0987	0.5989 [0.0000; 0.9572]	42.6% [11.4%; 62.9%]	13	0.1238	0.0808	0.1849	0.0181

Supplementary Table 3: Estimates of all outcomes of interest evaluated in the immunosuppressed CYP vs CYP in general population evaluated by our meta-analysis. The presence of reporting bias would be supported by a larger number of 'studies' added by "trim-and-fill", larger changes to the estimate following "trim-and-fill" and a p-value for the asymmetry test below 0.05. **Legend:** CI-confidence interval, CYP- children and young people; ITU- intensive therapy unit

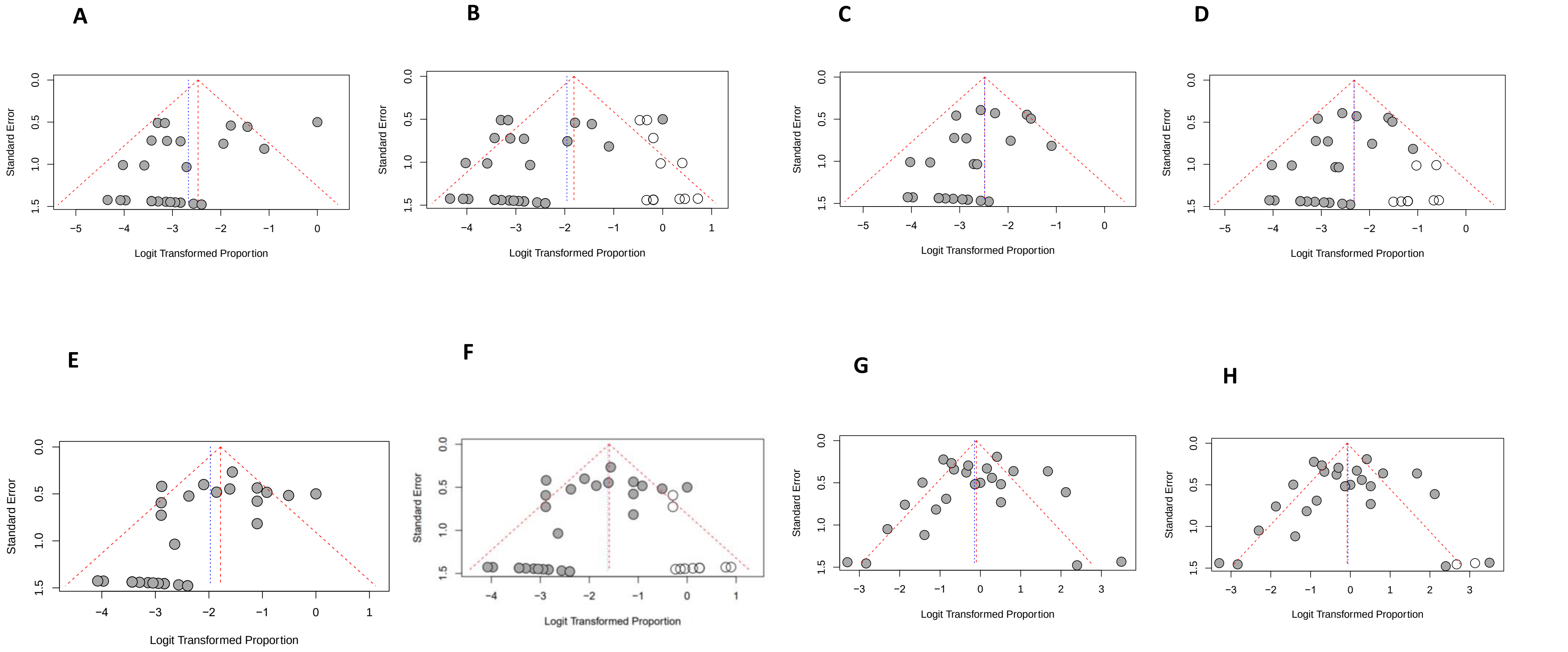

Supplementary Figure 1: Funnel plot for severe COVID-19 outcome studies in immunocompromised CYP

A. Funnel plot for death in immunocompromised CYP. B. Funnel plot following ‘trim-and-fill’ for death in immunocompromised CYP.

C. Funnel plot for invasive ventilation in immunocompromised CYP. Funnel plot following ‘trim-and-fill’ for invasive ventilation in immunocompromised CYP

E. Funnel plot for ITU admission in immunocompromised CYP. F. Funnel plot following ‘trim-and-fill’ for ITU admission in immunocompromised CYP

G. Funnel plot for hospitalisation in immunocompromised CYP. H. Funnel plot following ‘trim-and-fill’ for hospitalisation in immunocompromised CYP.

Open circles indicate added ‘studies’ following “trim-and-fill”. The number of added ‘studies’ may be indicative of the extent of reporting bias.

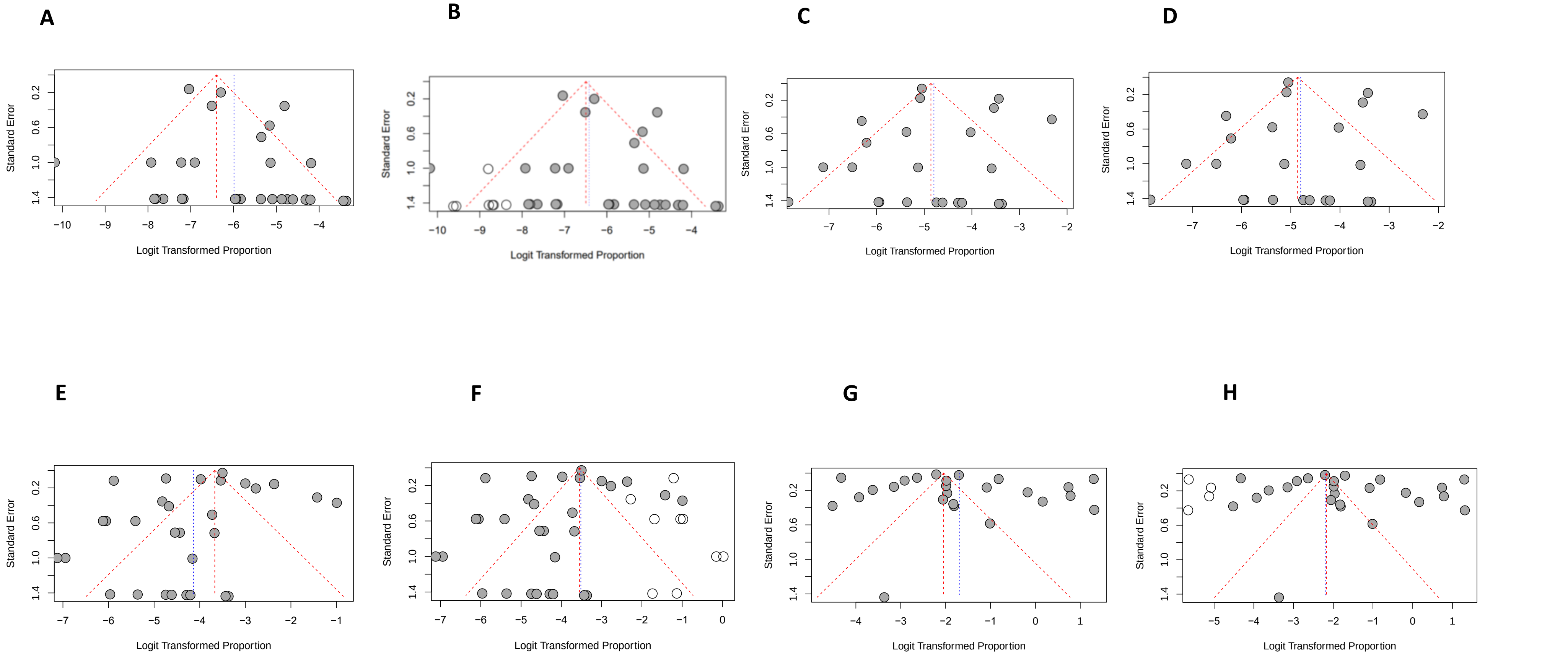

Supplementary Figure 2: Funnel plot for severe COVID-19 outcome studies in CYP in general population

A. Funnel plot for death in in CYP in general population. B. Funnel plot following ‘trim-and-fill’ for death in CYP in general population.

C. Funnel plot for invasive ventilation in CYP in general population. D. Funnel plot following ‘trim-and-fill’ for invasive ventilation in CYP in general population

E. Funnel plot for ITU admission in CYP in general population. F. Funnel plot following ‘trim-and-fill’ for ITU admission in CYP in general population.

G. Funnel plot for hospitalisation in in CYP in general population. H. Funnel plot following ‘trim-and-fill’ for hospitalisation in CYP in general population.

Open circles indicate added ‘studies’ following “trim-and-fill”. The number of added ‘studies’ may be indicative of the extent of reporting bias.
